# Annotations

**Published:** 1901-02-02

**Authors:** 


### Annotations.

### Shell Fish and Enteric Fever.

The recent occurrence, in the St. Pancras district, of a considerable number of cases of enteric fever, which appeared on investigation by Dr. Sykes to be attributable to the consumption of mussels, brings us back once more to the dangers which must continue to attend the use of shell fish as food so long as Parliament chooses not to intervene for the protection of the mussel, oyster, and other "beds" from contamination with sewage matter. It appears that in this particular case the incriminated mussels were traced to a place in Holland, and this illustrates the real difficulty which stands in the way of effective legislation for the protection of this department of our food supply, namely, that a considerable portion of it comes from places abroad over which we can of course exercise no jurisdiction. We are not sure, however, that although we can exercise no jurisdiction we might not bring a very considerable pressure to bear towards the improvement of the sanitary surroundings of some of the more important foreign sources of supply. The English trade is one of great importance to these places, and if, by dint of careful legislation for the control and protection of our breeding grounds, the safety and harmlessness of English shell fish were once assured, we cannot but think that the foreigners, for the sake of retaining a valuable market, would soon be led to impose similar regulations on their own fisheries. Unfortunately we do not come clean-handed to the discussion of the matter. There is plenty of good and wholesome shell fish to be obtained from English waters, but then there is quite enough that is of doubtful origin, that is to say, that is grown and stored amid conditions inimical to safety, to prevent the development of any special preference for the English product.

### Municipal Bakeries.

"A municipality, after all, is but a corporate association of bread-consumers. Why should it not produce what it consumes?" This is the conundrum propounded in one of the latest of the tracts issued by the Fabian Society, and it is just like the pretty little ways of that society to end its leaflets by such a "poser." For, indeed, why not? We are by no means in love with the municipalisation of anything, except, perhaps, water, gas, tram-lines, telephones, and other arrangements which involve the tearing up of the public streets; but if a case could be made out for any industry, and if it could fairly be claimed that any form of manufacture should be taken in hand by the municipality and worked for the good of all, we cannot doubt that the very first to be so dealt with would be the making of bread—the staff of life, the article of diet which more than any other is partaken of by all; the one which forms the basis of the food of the majority, and the one which, perhaps, of all others, is prepared in the least satisfactory manner, and amid the most unsatisfactory surroundings. According to "Fabian Tract No. 94," of 82 bakehouses in Fulham, 70 are still underground; as are 47 of the 57 in Chelsea, 40 of the 61 in Hammersmith, 63 of the 88 in Shoreditch, and 25 of the 27 in St. James's, Westminster. For years back sanitarians have been hammering away at this question, and proclaiming the evils connected with the manner in which, and the places in which the ordinary bread of the people is prepared. Dr. Waldo has made himself especially prominent in the crusade. His book "Bread, Bakehouses and Bacteria" is a formidable indictment of the state of affairs existing at the time it was written, and it is clear from the report of the medical officer of health for Manchester for the year 1898, that in other towns much the same conditions are to be met with as were so graphically described by Dr. Waldo. But to come back to the question of municipalisation. There would be no reason or excuse for the municipalities intervening if private enterprise would do the work. At present, however, it does not make much sign. Here and there we find a large factory established where with good machinery and modern appliances bread is made in a cleanly manner which is entirely satisfactory, but speaking generally private enterprise seems to run in the direction of the small baker, whose little speculative bakery too often becomes, we are told, the "tied house" of some great miller. This is a great evil. If private enterprise would establish great bakeries in which good wholesome bread could be made in a good wholesome fashion, we would gladly keep all municipalisation at bay, trusting to competition to keep prices down, and to the sanitary authorities to keep quality up. But if competition is to be undermined by the bakeries falling into the hands of the millers, and if inspection is to be rendered well nigh impossible from the multitude of little places where bread is baked, the outlook takes on a different aspect and we might perhaps be tempted even to shake hands with our imaginative friends the Fabians.

### The Extemporaneous Sterilisation of Water.

At the last meeting of the Epidemiological Society, Dr. Louis Parkes and Dr. Samuel Rideal brought forward a suggestion for the extemporaneous sterilisation of water, or at least for removing from it the micro-organism which is most fatal to armies in the field—namely, the bacillus typhosus. Their aim was to find some means other than boiling or filtering by which to destroy the virulence or inhibit the growth of this organism, whilst not rendering the water unpalatable or injurious to the consumer. Of course the ordinary antiseptics are out of the question, But as the result of numerous experiments it was found that by the addition of a small quantity of sodium bisulphate to boiled water infected with measured quantities of broth cultures of bacillus typhosus the mixture was quickly rendered sterile. The authors felt justified in assuming (1) that water naturally infected with the virus of the disease does not contain so very large a proportion of the bacillus; (2) that this organism is not by any means resistant to acids or other weak disinfectants; and (3) that the use of sodium bisulphate as described has possibly a future before it, more especially where sterilisation by boiling and filtration are not readily available. They recommend that the sodium bisulphate should be employed in the form of tabloids, each containing five grains, and so made as to dissolve quickly. Three of these should be added to each pint of water, which should then be allowed to stand for fifteen minutes, at the end of which time it is hoped that it will be sterilised. It sounds very simple, but we are afraid it is too good to be true.

